# A Cascaded MEMS Amplitude Demodulator for Large Dynamic Range Application in RF Receiver

**DOI:** 10.3390/mi12121515

**Published:** 2021-12-05

**Authors:** Hao Yan, Xiaoping Liao, Chenglin Li, Chen Chen

**Affiliations:** 1 National ASIC Research Center, Southeast University, Nanjing 210096, China; 2 Key Laboratory of MEMS of the Ministry of Education, Southeast University, Nanjing 210096, China; yee_dream@seu.edu.cn (C.L.); chchen@seu.edu.cn (C.C.)

**Keywords:** AM demodulator, microelectromechanical systems (MEMS), capacitive, thermoelectric

## Abstract

An amplitude demodulator with a large dynamic range, based on microelectromechanical systems (MEMS), is proposed in this paper. It is implemented as a cascade of a capacitive and a thermoelectric sensor. Two types of the transducer can improve the measurement range and enhance the overload capacity. This MEMS-based demodulation is realized by utilizing the square law relationship and the low-pass characteristic during the electromechanical and thermoelectric conversion. The fabrication of this device is compatible with the GaAs monolithic microwave integrated circuit (MMIC) process. Experiments show that this MEMS demodulator can realize the direct demodulation of an amplitude modulation (AM) signal with a carrier frequency of 0.35–10 GHz, and cover the power range from 0 to 23 dBm. This MEMS demodulator has the advantages of high power handling capability and zero DC power consumption.

## 1. Introduction

Modern communication systems generate a huge demand for a large-scale manufacturing process, low power consumption, miniaturization, and new functionalities with various electronic components. The classical amplitude demodulator for the radio-frequency (RF) heterodyne receiver is composed of a local oscillator (LO), a mixer, and a low-pass filter (LPF) [1,2]. Recent advances in micro-electromechanical system (MEMS) techniques provide new solutions for signal demodulation [3]. Researchers have focused on the improvement of MEMS electrostatic [4,5,6,7,8] or thermal [9,10,11,12] mixer-filters to replace the traditional mixer and low-pass filter (LPF) in RF receivers. To acquire a higher quality of factor and sensitivity, they attempt different approaches, including mechanism analysis, structure optimization, and process/material improvement [13,14,15,16,17,18]. The use of those MEMS mixer-filters can not only replace separate components to reduce the size of communication devices, but also serve as a passive device with low power consumption. Despite these successes, the engineering work still needs to be addressed [18,19]. Firstly, the carrier frequencies of these devices are generally less than the GHz range, as their characteristic impedances are larger than 50 ohms. Secondly, to ensure higher sensitivity, those resonators have a high DC bias voltage. They require voltage booster circuits, which leads to the incurrence of extra cost and the consumption of additional power.

Further, Yan et al. propose a novel on-line MEMS-based amplitude demodulator. Instead of the mechanical resonator and the piezoelectric transducer, they apply thin-film thermopiles to realize the thermoelectric conversion with a low-pass characteristic [20]. This design facilitates the achievement of impedance matching, and the carrier frequency can be increased to 10 GHz. In addition, the thermoelectric transducer requires no DC bias voltage. However, this type of device suffers from thermal reliability-related issues. Its sensitivity is reduced due to deterioration of the thermal conductivity and the Seebeck coefficient at around 30 K. Additionally, a thinner heat transfer structure is commonly adopted to improve the sensitivity [21,22]. Moreover, a high-power handling capability is one of the key parameters for radar front-end transceiver systems or 5G applications [23,24]. These factors exacerbate the heat emission issue of the micro structure.

**Figure 1:**
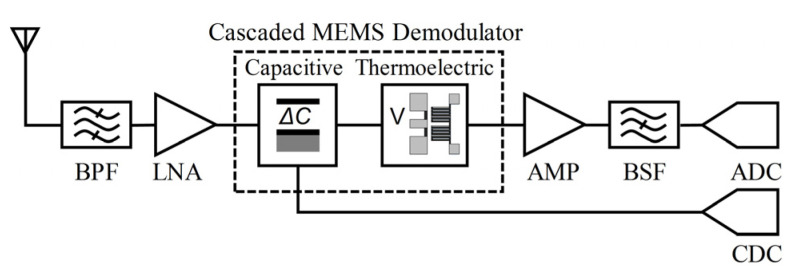
MEMS-based demodulator is a part of a radio receiver.

To overcome this problem, a cascaded MEMS amplitude with a thermopile-type and capacitive-type sensing structure, to expand the dynamic range, is proposed in this paper. The cascaded design of the power sensor can realize an improved dynamic range and high sensitivity [25]. The principle of the signal demodulation is analyzed based on an equivalent circuit model. The structure of this device is optimized by the simulation. Experiments show that it can realize the direct demodulation for (0.35–10 GHz) RF signals and cover the power range of 0–23 dBm. This novel cascaded design has the potential to expand the dynamic range applied in RF receivers. As Figure 1 shows, the system is composed of an antenna, a band-pass filter (BPF), a low noise amplifier (LNA), a MEMS demodulator detector, a DC amplifier (AMP), a band-stop filter (BSF), an analog-to-digital converter (ADC), and a capacitor-to-digital converter (CDC). This proposed architecture for the RF receiver not only realizes a direct conversion from a modulated RF signal to a baseband signal without the local oscillator (LO) but can also serve as a passive sensing device with zero DC power consumption.

## 2. Principle and Design

**Figure 2:**
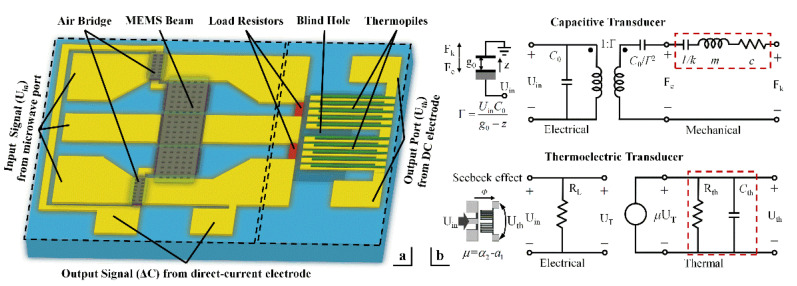
Cascaded MEMS demodulator: (**a**) schematic overview and (**b**) equivalent circuit.

A schematic overview of the cascaded MEMS demodulator is shown in Figure 2a. It comprises an on-line capacitive transducer and a thermoelectric transducer. A MEMS beam, a section of coplanar waveguide (CPW), two measuring electrodes, and a dielectric layer compose a plane-parallel capacitor. As the microwave power is applied to the sensor, part of the RF signal is coupled to the MEMS beam. The MEMS beam can respond to the DC component of the RF signal and displacement occurs due to the electrostatic force. Two measuring electrodes below the beam sense this micro displacement by detecting the change in capacitance. Two air bridges are designed to interconnect the CPW ground lines. Load resistors absorb the microwave power and convert it into heat. The thermopiles sense the temperature difference and output a DC thermoelectric voltage through two electrodes, based on the Seebeck effect [21,22]. Two blind holes are designed under the thermopiles to improve the thermoelectric conversion performance.

Figure 2b explicitly equates the actual MEMS demodulator to an equivalent circuit in the sub-circuit, to enable the electro-mechanical conversion to occur. The mechanical and thermal parameters can be equivalent to the electrical quantities; the mechanical force *Fk* and displacement *z* are equivalent to the voltage (*U_F_*) and current (*I_d_*), respectively. The stiffness, mass, and damping correspond to an electrical capacitor (*1*/*k*), electrical inductor (*m*), and resistors (*c*), respectively, and depend on the material characteristics and the device structure [4,5]. Specifically, the electromechanical transduction can be simulated by a transformer, and the thermoelectric transduction can be treated as a voltage-controlled voltage source (VCVS). Correspondingly, for the electro-thermal conversion, the temperature difference and heat flux are equivalent to the voltage (*U_T_*) and current (*I_h_*). The heat capacity, thermal conduction, and thermal convection losses correspond to the electrical capacitor (*C_th_*) and resistors (*R_th_*). As the load resistor (*R*_load_ = 50 ohms) is determined, the temperature difference (*U_T_*) and the mechanical force (*U_F_*) are both proportional to the input voltage squared. Due to this square law, the output response of the system is the product of the input signal. When the input signal is an amplitude modulation signal, *U_in_* = *U_a_* [1 + *m_a_* × cos(Ω*t*)] cos(*ω_c_t*), the input voltage squared can be expressed as follows:

$$\begin{array}{l} U_{in}^{2}=\frac{1}{2}(1+\frac{m_{a}^{2}}{2})U_{a}^{2}+m_{a}U_{a}^{2}(\cos\Omega t+\frac{m_{a}}{4}\cos2\Omega t)\\ +\frac{m_{a}^{2}}{8}U_{a}^{2}\left[\cos2\left(\omega_{c}+\Omega \right)t+\cos2\left(\omega_{c}-\Omega \right)t\right]\\ +\frac{m_{a}}{2}U_{a}^{2}\left[\cos\left(2\omega_{c}+\Omega \right)t+\cos\left(2\omega_{c}-\Omega \right)t\right]\\ +\frac{1}{2}(1+\frac{m_{a}^{2}}{2})U_{a}^{2}\cos2\omega_{c}t \end{array}$$

where *U_a_* is the amplitude, *m_a_* (0 < *m_a_* < 1) is the modulation depth, *ω_c_* = 2π*f_c_* is the frequency of the carrier wave, and Ω = 2π*f_m_* is the frequency of the modulation waveform. Moreover, the frequency response of two sensors is much lower than the RF bands. High-frequency components (2*ω_c_*, 2*ω_c_* ± Ω, 2*ω_c_* ± 2Ω), after multiplication, can be filtered out as a result [20]. Generally, the *m_a_*cos(2Ω*t*)/4 is much less than one and can be ignored. Therefore, two types of power sensors can both demodulate AM signals.

**Figure 3:**
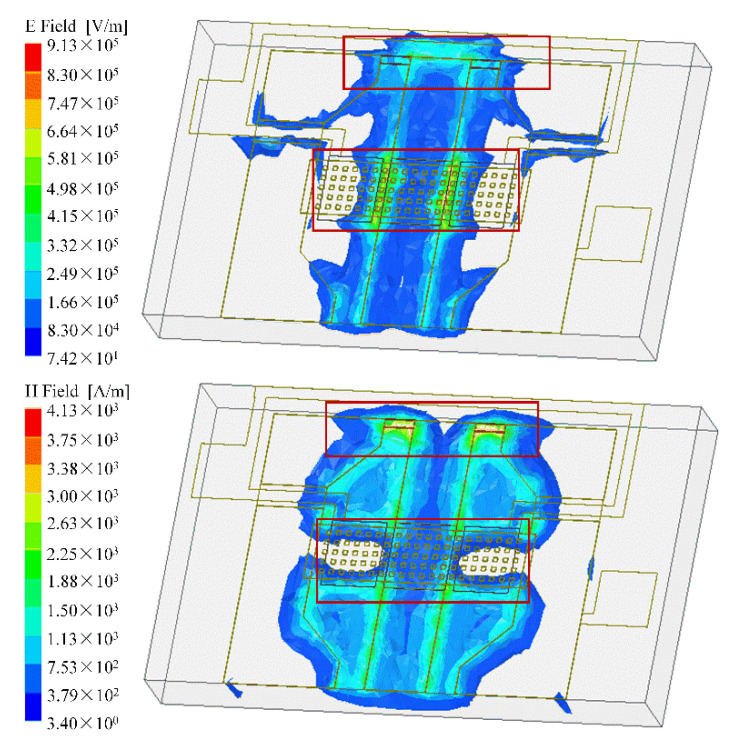
Electric and magnetic field distribution of cascaded MEMS demodulator simulated by Ansys HFSS.

**Figure 4:**
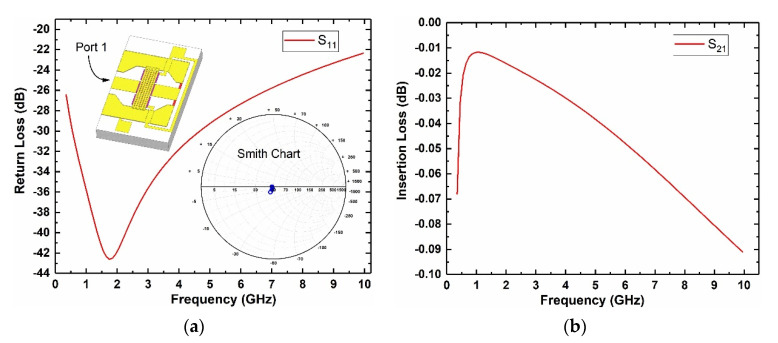
Microwave performance: (**a**) MEMS demodulator return losses S_11_; (**b**) capacitive transducer insertion loss S_21_.

The cascaded MEMS demodulator is designed to operate up to 10 GHz. The structure of this device is simulated by ANSYS HFSS (high-frequency structure simulator) to determine the size parameters. Figure 3 shows the surface electric and magnetic field distribution of the MEMS transducer at 10 GHz. The electromagnetic field intensity distribution shows that the RF signal is concentrated in the following two areas, as marked in the red rectangle of Figure 3: (i) the overlapping area between the MEMS beam and the signal line; (ii) around the thermal resistor. This indicates that the two areas are the effective sensing regions, which can absorb the principal microwave signal and transform it into a low-frequency signal, as the temperature difference (*U_T_*) and the mechanical force (*U_F_*). Figure 4 shows that the return loss of port 1 (S_11_) > 22 dB at 0.35–10 GHz, and the insertion loss of the capacitive transducer is less than 0.1 dB at different frequencies. The inset Smith chart in Figure 4a indicates that the impedance achieved matches 50 ohms at the operation frequency.

## 3. Fabrication

**Figure 5:**
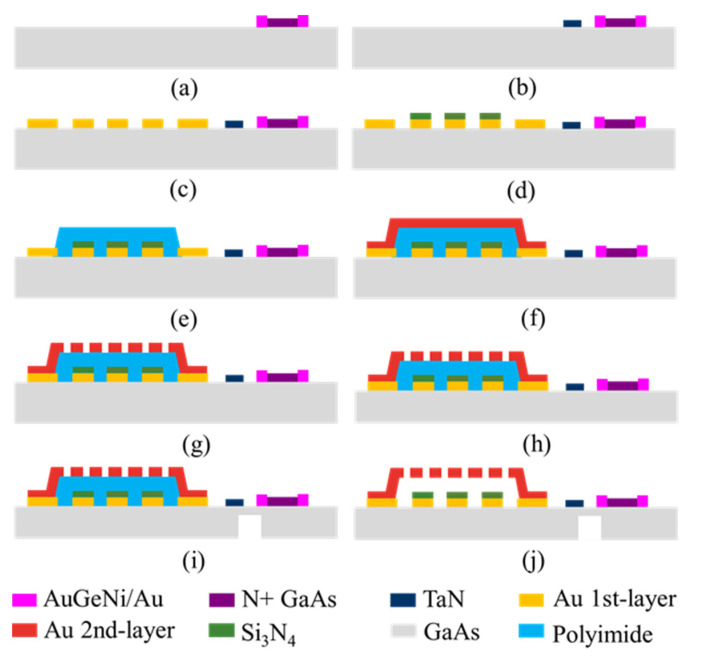
(**a**–**j**) Process steps of the cascaded MEMS amplitude demodulator.

The cascaded MEMS demodulator is fabricated by the GaAs MMIC process. The fabrication steps are shown in Figure 5a–j and listed as follows.

(a) Doping N + GaAs of 1 × 10^18^ cm^−3^ to form the thermopile;
(b) Sputtering a TaN layer, and forming the isolation resistors and terminal resistors;
(c) Evaporating a 0.3-micrometer-thick Au layer, and lifting off to form the CPW transmission lines and electrodes;
(d) Depositing a 0.23 μm Si_3_N_4_ layer by PECVD as a dielectric layer between the MEMS bridge and the CPW lines;
(e) Depositing a 1.6 μm polyimide layer by PECVD as a sacrificial layer;
(f) Evaporating a 50/150/30-nanometer-thick Ti/Au/Ti layer as the seed layer, and electroplating a 2 μm Au layer as the top metal layer;
(g) Lifting off the Au layer to form the hole array pattern;
(h) Thinning the substrate to 100 μm;
(i) Etching the substrate underneath the load resistors and thermopiles to 20 μm;
(j) Removing the sacrificial layer and releasing the MEMS beam to form the movable microstructure.

**Figure 6:**
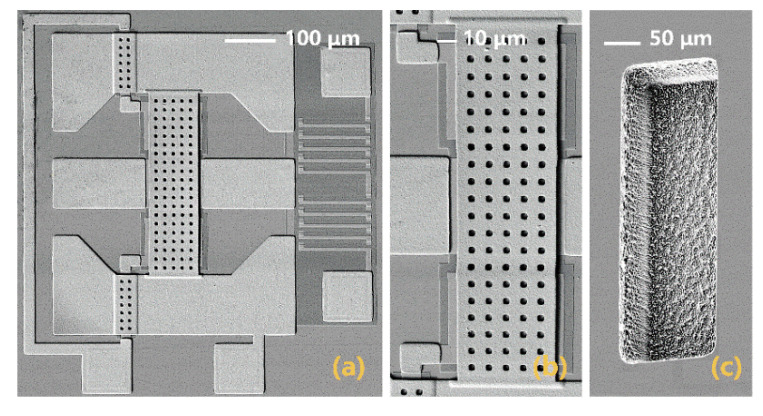
SEM image: (**a**) cascaded MEMS demodulator; (**b**) MEMS clamped-clamped beam and (**c**) backside-etch structure and blind hole.

Figure 6a shows the SEM image of the cascaded MEMS demodulator. The separate insets show the MEMS clamped–clamped beam and the rectangular blind hole. The corresponding structural parameters are also listed in Table 1.

## 4. Measurement

**Figure 7:**
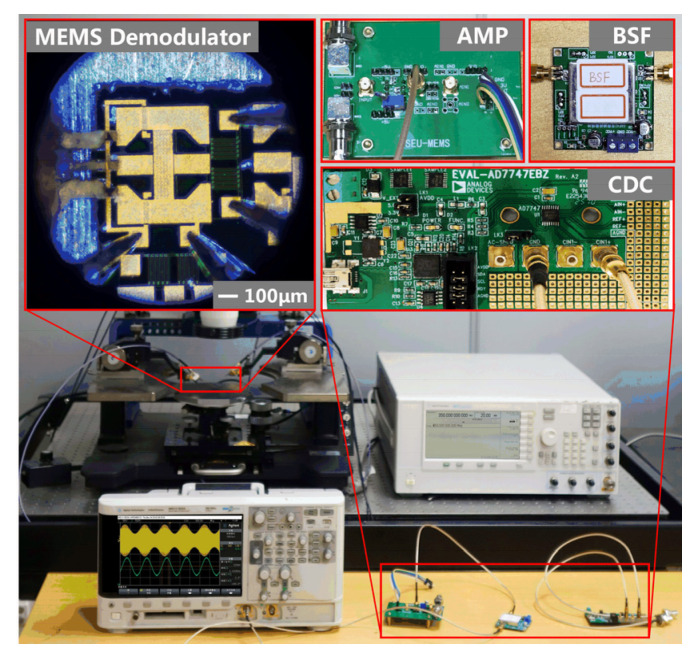
Test setup and inset: MEMS demodulator and its processing circuit.

**Figure 8:**
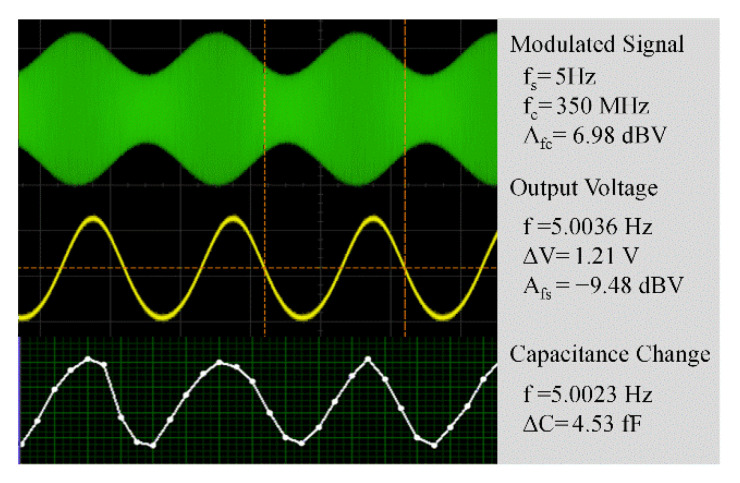
Cascaded MEMS-based demodulation of an AM signal.

The measurement system includes a microwave probe station, an RF signal generator, an oscilloscope, and signal processing circuits, as shown in Figure 7. The inset shows the connection mode of the probes. A capacitance-to-digital converter (CDC) is applied to measure the capacitance change (Δ*C*) of the detector. A band-stop filter (BSF) is adopted to reduce the industrial-frequency noise. The demodulation results of an AM signal are shown in Figure 8. The carrier wave’s frequency (*f_c_*) of the AM signal is 0.35 GHz, and the modulation wave’s frequency is 5 Hz. The signal power is 20 dBm and the modulation depth (*m_a_*) is 0.2. The results show that the output curves accord well with the envelope signal, which proves the validity of the MEMS-based demodulation principle.

**Figure 9:**
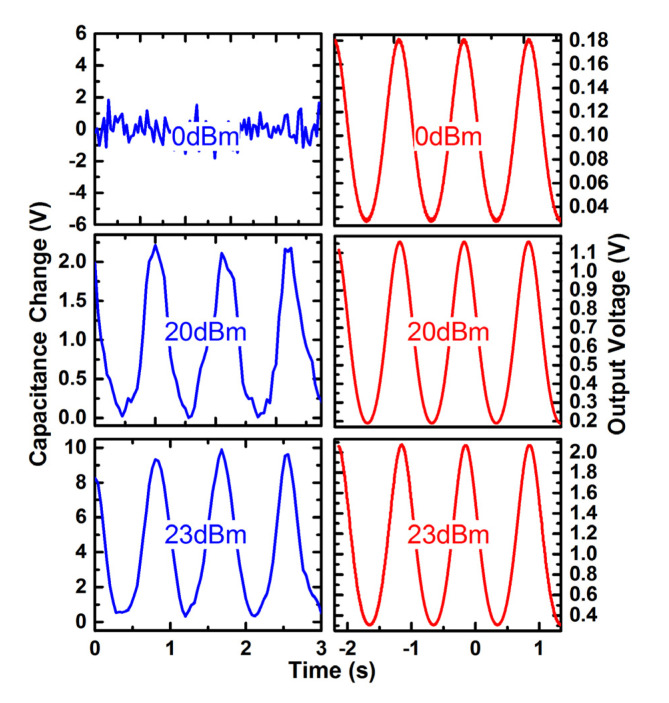
Output response of the MEMS demodulator under low—(0 dBm), medium—(20 dBm) and high-power—(23 dBm) AM signal (*fm* = 1 Hz, *fc* = 10 GHz).

**Figure 10:**
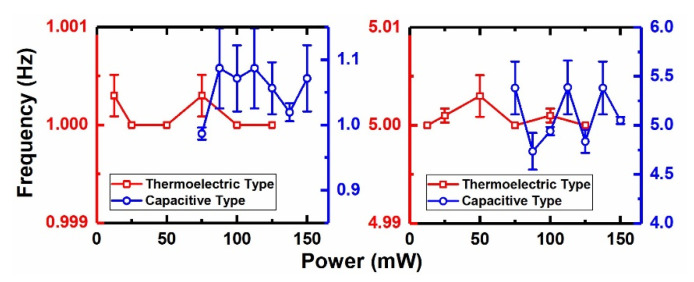
Different frequency’s test errors versus different input power.

Figure 9 shows the output waveform of the MEMS demodulator under a low-, medium- and high-power (0, 20 and 23 dBm) AM signal (*f_m_* = 1 Hz, *f_c_* = 10 GHz, *m_a_* = 0.2). Figure 10 shows the frequency’s test errors versus the different power levels, and the maximum relative error is less than 8%. The experiments show that the two types of power sensors that are cascaded in this demodulation system can realize the RF signals’ amplitude demodulation with a carrier frequency of 0.35–10 GHz, and cover the power range of 0–23 dBm (1–200 mW).

## 5. Conclusions

This paper has outlined a novel passive device based on two types of MEMS power sensors for RF signal direct-conversion amplitude demodulation. The experiments show that the two types of sensors that are cascaded in this system can realize the direct demodulation for (0.35–10 GHz) RF signals and cover the power range of 0–23 dBm. Therefore, this MEMS-based demodulator has the advantage of high power handling capability. Moreover, the fabrication of this device is compatible with the GaAs MMIC process. The MEMS-based demodulator with zero DC power consumption has potential for microwave communication applications, particularly IoT and sensor node applications, which have strict requirements for volume and consumption.

## Figures and Tables

**Table 1:** Structural parameters for cascaded MEMS amplitude demodulator.

Quantity	Value
Width of main CPW signal line	100 μm
Gap between ground and CPW signal line	58 μm
Width of ground line	300 μm
Width of the clamped beam	100 μm
Length of the clamped beam	400 μm
Height of the air-gap	1.6 μm
Size of the thin-film resistor	14.5 μm × 58 μm
Size of the air bridge	120 μm × 40 μm
Thickness of dielectric layer	0.23 μm
Thickness of ground & CPW signal line	2.4 μm

## References

[1] Mirabbasi S., Martin K. (2000). Classical and modern receiver architectures. IEEE Commun. Mag..

[2] Razavi B. (1997). Design considerations for direct-conversion receivers. IEEE Trans. Circuits Syst. II Analog. Digit. Signal Process..

[3] De Los Santos H.J., Richards R.J. (2001). MEMS for RF/Microwave wireless applications: The next Wave-Part II. Microw. J..

[4] Wong A.C., Nguyen C.T.C. (2004). Micromechanical mixer-filters (“mixlers”). J. Microelectromech. Syst..

[5] Koskenvuori M., Tittonen I. (2007). Improvement of the conversion performance of a resonating multimode microelectromechanical mixer-filter through parametric amplification. IEEE Electron Device Lett..

[6] Bartsch S.T., Rusu A., Ionescu A.M. (2012). A single active nanoelectromechanical tuning fork front-end radio-frequency receiver. Nanotechnology.

[7] Chung S.R., Park S., Abdel-Rahman E.M., Yeow T., Khater M. (2013). Architecture for MEMS-based analogue demodulation. J. Micromech. Microeng..

[8] Liu R., Nilchi J.N., Li W.C., Clark T.C. Soft-impacting micromechanical resoswitch zero-quiescent power AM receiver. Proceedings of the 2016 IEEE 29th International Conference on Micro Electro Mechanical Systems (MEMS).

[9] Reichenbach R.B., Zalalutdinov M., Aubin K.L., Rand R., Houston B.H., Parpia J.M., Craighead H.G. (2005). Third-order intermodulation in a micromechanical thermal mixer. J. Microelectromech. Syst..

[10] Reichenbach R.B., Zalalutdinov M., Parpia J.M., Craighead H.G. (2006). RF MEMS oscillator with integrated resistive transduction. IEEE Electron Device Lett..

[11] Mastropaolo E., Gual I., Cheung R. (2010). Silicon carbide electrothermal mixer-filters. Electron. Lett..

[12] Svilicic B., Mastropaolo E., Flynn B., Cheung R. (2012). Electrothermally actuated and piezoelectrically sensed silicon carbide tunable MEMS resonator. IEEE Electron Device Lett..

[13] Erismis M.A. (2018). A micromechanical analogue mixer with dynamic displacement amplification. Int. J. Electron..

[14] Krakover N., Maimon R., Tepper-Faran T., Gerson Y., Rand R., Krylov S. (2019). Mechanical superheterodyne and its use for low frequency vibrations sensing. J. Microelectromech. Syst..

[15] Chen Z., Kan X., Yuan Q., Wang T., Yang J., Yang F. (2020). A Switchable High-Performance RF-MEMS Resonator with Flexible Frequency Generations. Sci. Rep..

[16] Defoort M., Rufer L., Fesquet L., Basrour S. (2021). A dynamical approach to generate chaos in a micromechanical resonator. Microsyst. Nanoeng..

[17] Shao S., Gao A., Wang Y., Wu T. Wide bandwidth lorentz-force magnetometer based on lateral overtone bulk acoustic resonator. Proceedings of the 2021 IEEE 34th International Conference on Micro Electro Mechanical Systems (MEMS).

[18] Wei L., Kuai X., Bao Y., Wei J., Yang L., Song P., Wang X. (2021). The Recent Progress of MEMS/NEMS Resonators. Micromachines.

[19] Li W.C. Micromechanical vibro-impact resonator-enabled sensing applications. Proceedings of the 2021 21st International Conference on Solid-State Sensors, Actuators and Microsystems (Transducers).

[20] Yan H., Liao X., Li C. (2017). A Dual-Channel MEMS Amplitude Demodulator for On-Line Detection in Radio Relay Station. IEEE Electron Device Lett..

[21] Yi Z., Liao X. (2016). A 3D model of the thermoelectric microwave power sensor by MEMS technology. Sensors.

[22] Zhang Z., Ma Y. (2018). DC-25 GHz and low-loss MEMS thermoelectric power sensors with floating thermal slug and reliable back cavity based on GaAs MMIC technology. Micromachines.

[23] Schmid U., Reber R., Schuh P., Opperman M. Robust wideband LNA designs. Proceedings of the 2014 9th European Microwave Integrated Circuit Conference.

[24] Rudolph M. GaN HEMTs for low-noise amplification—Status and challenges. Proceedings of the 2017 Integrated Nonlinear Microwave and Millimetre-wave Circuits Workshop (INMMiC).

[25] Yi Z., Yan H., Liao X. (2017). Theoretical and Experimental Investigation of Cascaded Microwave Power Sensor. IEEE Trans. Electron Devices.

